# Graphene promotes the growth of *Vigna angularis* by regulating the nitrogen metabolism and photosynthesis

**DOI:** 10.1371/journal.pone.0297892

**Published:** 2024-03-07

**Authors:** Jun Qiao, Zhiwen Chen, Jianguo Zhao, Jing Ren, Hao Wang, Caiyan Zhi, Jingwei Li, Baoyan Xing, Hui Nie

**Affiliations:** 1 Engineering Research Center of Coal-based Ecological Carbon Sequestration Technology of the Ministry of Education, Shanxi Datong University, Datong, China; 2 Key Laboratory of Graphene Forestry Application of National Forest and Grass Administration, Shanxi Datong University, Datong, China; 3 School of Chemistry and Chemical Engineering, Shanxi Datong University, Datong, China; Abdul Wali Khan University Mardan, PAKISTAN

## Abstract

Graphene has promising applications in agriculture and forestry. In the current study, six different concentrations of graphene (0mg/L, 0.01mg/L, 0.10mg/L, 1.00mg/L, 10.00mg/L, and 100.00mg/L) were used to investigate its effect on the growth and development of *V*. *angularis* plants in soil culture. The results showed that the group treated with 1.00mg/L graphene (G-1) had significantly increased plant height (19.86%), stem diameter (24.33%), and leaf area (13.69%), compared to the control group (CK). Moreover, all concentrations of graphene had positive effects on the total root length, total root surface area, and the number of root tips of *V*. *angularis*. Compared to the CK group, the G-1 group had significantly increased leaf water potential (37.89%), leaf conductivity (2.25%), and SOD, POD, and CAT activities (47.67%, 35.22%, and 199.3%, respectively). The G-1 group also showed improved leaf net photosynthetic rate, chlorophyll content, and soluble sugar content (51.28%, 24.25%, and 38.35%, respectively), compared to the CK group. Additionally, 1.00mg/L graphene led to a 23.88% increase in the podding rate and a 17.04% increase in the yield of *V*. *angularis* plants. The rhizosphere soil of *V*. *angularis* treated with 1.00mg/L graphene had a 25.14% increase in hydrolyzable nitrogen content and a 66.67% increase in available phosphorus content. RNA-seq data indicated that 1.00mg/L graphene induced the expression of photosynthesis and nitrogen transmembrane transport genes, including ATP synthase subunit b, photosystem I reaction center subunit XI, photosystem I reaction center subunit IV A, ferredoxin, and psbP-like protein 1, as well as genes for photosynthesis antenna proteins, glutamine synthetase, glutamate dehydrogenase 1, cyanate hydratase, protein fluG-like, and NRT1/PTR family, suggesting that graphene promoted the growth and development of *V*. *angularis* by enhancing the photosynthesis and nitrogen metabolism processes in *V*. *angularis* plants. Our results indicated that a suitable concentration of graphene could significantly promote the growth of *V*. *angularis* plants in soil.

## Introduction

Carbon is the fundamental element for the formation of all living organisms on Earth. It is the basic unit of life [1–3]. Graphene is composed of one carbon atom and three adjacent carbon atoms, and sp^2^ hybridization occurs between each carbon atom in S hybrid orbital, Px orbital and Py orbital, forming a hexagonal honeycomb network structure of two-dimensional crystal material [4]. Carbon atoms are combined by covalent bonds, each carbon atom shares electrons with three adjacent carbon atoms to form three sigma bonds, the adjacent bond forms a bond Angle of 120°, the bond length is 0.142 nm, the structure is very stable, and the remaining outermost electron is shared by four carbon atoms to form a π bond perpendicular to the graphene plane [5–8]. Graphene is an exceptional engineered nanomaterial with significant potential due to its large surface area, remarkable electrical conductivity, mechanical strength, and thermal conductivity [9–11].

Graphene Family Nanometer materials (GFNs) as a typical representative of planar carbon nanomaterials, and it includes single-layer graphene, multi-layer graphene, ultra-thin graphite, graphene oxide, reduced graphene oxide and graphene nanosheets and other nanocarbon materials [12]. Since graphene was discovered in 2004, experts and scholars have conducted extensive research on graphene nanomaterials. In recent years, researchers have become increasingly interested in studying the impact of GFNs on plant growth and development.

Most of the research conducted so far has been focused on the impact of graphene on plant physiological parameters and phenotypic variations. For instance, a study by Zhang et al. [13] investigated the effects of graphene oxide on the seed germination and seedling growth of tomatoes. The study found that low concentrations of graphene could increase the germination rate of tomato seeds, accelerate the germination process, and increase the rhizome length of seedlings compared to the control group, but decrease the biomass. Meanwhile, Jiao et al. [14] explored the effect of graphene oxide on tobacco seedlings, and discovered that the effect of graphene oxide on tobacco seedlings was related to the treatment time. After 20 days of treatment, the root of tobacco in the experimental group became shorter, while after 35 days of treatment, the adventitious root of tobacco increased by two to three times compared with the control group, and the fresh root weight also increased slightly. Additionally, the activity of antioxidant enzymes such as SOD and POD increased, while the content of MDA decreased.

In other studies, He et al. [15] used the unique structural properties of graphene oxide, which has both hydrophilic and hydrophobic groups, to investigate its effect on spinach and chives. They discovered that a low amount of graphene oxide improved the emergence rate of both plants. The hydrophilic groups in graphene oxide can prevent water loss from the soil, while the hydrophobic groups can promote water absorption into the seed, which enhances seed germination [15]. Similarly, Chen et al. [16] found that a modified graphene treatment of 5 g/kg significantly increased the fresh and dry weight of alfalfa, and also improved the chlorophyll content and antioxidant enzyme activity of the plant leaves. Additionally, Zhao et al. [17] found that the enriched functional groups on the surface of graphene could help plants absorb more macronutrients from the medium. Furthermore, a recent study showed that an appropriate amount of graphene oxide promoted the length of roots and shoots, the number of leaves, root nodules per plant, the number of pods, and the seeds per pod of mungbean plants [18].

Despite many researchers have conducted studies on the effects of graphene on plant growth, but few studies provided the molecular mechanisms that explored the graphene interaction with Leguminosae plant. *V*. *angularis* (*Vigna angularis*) is a crop enriched in nutritional value, and often cultivated in adversity land of northern China. In current study, *V*. *angularis* was used to study the effects of graphene on the growth and development in soil. Low concentrations of graphene promoted the growth and development of *V*. *angularis* by regulating the photosynthesis and biological nitrogen fixation processes. The major contents were focused on the growth morphology, root morphology, and photosynthetic physiology of *V*. *angularis*, so as to analyze and explore the regulation mechanism of graphene on its growth and development. Our results indicated that the use of graphene as a strategy would make it possible to accelerate plant growth and the significant increase in the plant biomass, which has potential application values in agriculture.

## Materials and methods

### Preparation and characterization of graphene

The graphene used in this study was prepared by high-frequency AC pulse method [19]. The size distribution of the graphene particles was measured in a Mastersizer 3000 (Malvern Instruments Ltd. UK). The characteristics of the graphene were analyzed using ultraviolet-visible and Raman spectroscopy (HORIBA, LabRAM HR Evolution). Raman spectra were obtained using a Renishaw inVia™ Qontor with a 532 nm excitation laser. The morphology of the graphene was examined using scanning electron microscopy (SEM; TESCAN MAIA 3 LMH) and transmission electron microscopy (TEM; TecnaiG2F20 S-TWIN TMP). The C/O ratio of graphene was determined by X-ray energy-dispersion spectroscopy (EDS) (OXFORD INSTRUMENTS; INCAx-act).

### Plant cultivation and graphene exposure treatment

Six concentrations of graphene were performed: 0 mg/L (CK), 0.01 mg/L (G-0.01), 0.10 mg/L (G-0.1), 1.00 mg/L (G-1), 10.00 mg/L (G-10), 100.00 mg/L (G-100). Similar-sized *V*. *angularis* seeds were divided into six groups (36 seeds in each group), germinated in potting soil (12 pots for each concentration) in a growth chamber with three biological replications, and the resulting seedlings were maintained in a controlled environment at 28°C (day)/20°C (night) with a 16-h light/8-h dark photoperiod. The seeds were purchased from Jingdong Mall (https://item.jd.com/68539106063.html). The potting soil was composed of peat soil, perlite and vermiculite in a ratio of 3:1:1. Then, the potting soil was sterilized at 100 kpa and 121°C for 1 hour in an autoclave (LICHEN Technology, DGL-35B, China).

The *V*. *angularis* seedlings were watered with six different concentrations of graphene solutions once a week, starting from the sowing stage. After germination, the plants grown in soil pots were watered with solutions (1 L) containing varying concentrations of graphene each week. For the control group, the irrigation solution was the same amount of distilled water, and the other treatments were consistent with the experimental group. After 45 days of exposure to graphene, the roots of *V*. *angularis* were thoroughly washed with deionized water, dried with absorbent paper to remove the surface water, and then promptly frozen in liquid nitrogen. The frozen samples were stored at -80°C for RNA extraction.

### Root analysis of the *V*. *angularis* seedlings

The roots of *V*. *angularis* seedlings treated with various concentrations of graphene solution were analyzed for their architecture. After 45 days of exposure to graphene, the roots were thoroughly washed with deionized water and then scanned at 600 dpi using an Epson Perfection V850 Pro (Seiko Epson Corp., Tokyo, Japan). The images obtained were quantified with WinRHIZO (Version 4.0b, Regent Instruments Inc., Quebec, Canada) [20], which measured the total root length, total surface area, and number of root tips.

### Measurement of the POD, SOD and CAT activities in the plant roots

Antioxidant enzyme systems are a useful indicator of the damage sustained by plants in response to environmental stresses [21]. Plants have several antioxidant enzymes, including SOD, CAT, and POD, which are analyzed in response to different stressors. The activities of peroxidase (POD), superoxide dismutase (SOD), and catalase (CAT) were determined following previous methods [19, 21]. In brief, 3 mL of reaction solution, consisting of 50 mM PBS (pH 7.8), 13 μM methionine, 63 μM nitroblue tetrazolium (NBT), 1.3 μM riboflavin, and 30 μL of enzyme solution were incubated for 15 min under fluorescent light at 25°C. The reaction was then terminated immediately by shading, and the absorbance of the solution was subsequently measured at 560 nm. Correlation curves were constructed using the amount of enzyme solution as the x-axis and the relative percentage of inhibiting NBT photoreduction as the y-axis. The 50% inhibition rate of the enzyme solution was then determined as a SOD enzyme activity unit. POD activity was measured based on the oxidation of guaiacol, and the change in absorbance was recorded at 470 nm. In the 3 mL reaction mixture, 1 mL of 0.3% H_2_O_2_ (v/v), 0.95 mL of 0.2% guaiacol (w/v), 1 mL of pH 7.0 PBS, and 0.05 mL of enzyme solution was added to start the reaction. The rate of increase in OD was recorded at 470 nm, and an increase of 0.01/min was defined as a unit of peroxidase activity. For catalase (CAT) activity, in 3 mL of reaction solution, 1 mL of 0.3% H_2_O_2_ (v/v), 1.95 mL of H_2_O, and 0.05 mL of enzyme solution were added to start the reaction. The OD reduction rate was measured at a wavelength of 240 nm, and a reduction of 0.01/min was defined as one activity unit. To ensure accuracy, three biological replicates were performed for both the control and graphene groups to determine the POD, SOD and CAT activities.

### Measurement of plant height, stem diameter and leaf area

After being cultivated for 20 days with a graphene solution, the height of the plant was measured from the soil surface to the top of the plant using a steel ruler. The diameter of the stem below the cotyledon was measured using a vernier caliper. Five replicates were taken for each treatment. Additionally, the third leaf area located above the bottom of the plant was measured using a leaf area meter, and five replicates were measured for each treatment.

### Determination of leaf photosynthetic characteristics and leaf water potential

After 20 days of cultivation with graphene solution, net photosynthetic rate, stomatal conductance, intercellular CO_2_ concentration and transpiration rate, water use efficiency and other photosynthetic characteristics were determined by CIRAS-3 portable photosynthesometer of PP SYSTEMS (USA). The measurement time was from 8:30 a.m. to 11:30 a.m. Five replicates were measured for each treatment.

To measure the leaf water potential, the third leaves from the bottom of the plant were selected. The PSPPRO plant water potential system from WESCOR, INC, ENVIRONMENTAL PRODUCTS DIVISION, USA was used to measure the potential. During the test, the leaves were ground in a mortar to extract juice, and the juice was then put into a sample chamber with a filter paper of the same size. The sample was then saturated and moistened, and the water potential was measured. Three replicates were performed for each treatment.

### Determination of chlorophyll content, membrane permeability, soluble sugar content, and malonaldehyde (MDA) content

The chlorophyll content was extracted by direct extraction of acetone [22]. Briefly, 0.02 g *V*. *angularis* leaves were ground and added to a 5 mL EP tube along with 0.1 mL pure acetone and 2 mL 80% acetone solution. The tube was then extracted at room temperature until the leaf tissue turned white. The volume of 80% acetone was then fixed to 5 mL and centrifuged at 8000 rpm/min at 4°C for 10 min. The supernatant was then transferred to a clean EP tube and the absorbance was measured at 663 nm and 645 nm wavelengths, respectively. The cell membrane permeability was determined using the conductivity method [23]. The soluble sugar content was determined by the anthrone method [24]. The optimum conditions of anthrone sulfuric acid reaction system were as follows: the optimum anthrone dosage was 100 μL and concentrated sulfuric acid was 1,000 μL. The color reaction temperature was 100°C and the optimum reaction time was 7 min. The optimal extraction time was 10 min in boiling water bath. The malondialdehyde (MDA) content was determined by the thiobarbituric acid (TBA) assay [25]. 3 g of leaf sample was collected and ground in a mortar and pestle with liquid nitrogen. Then, 0.1 g leaf powder was added to a centrifuge tube containing 1 mL of 0.1% TCA and mixed by inverting the tube to precipitate the protein. The samples were then centrifuged at 12,000 rpm for 20 min and the supernatant was transferred to a new tube with 5 mL of 20% TCA. The mixture was boiled at 95 C for 15 min in a water bath and then quickly cooled on an ice bath for 10 min to stop the reaction. The mixture was then centrifuged at 12,000 rpm for 10 min and the supernatant was collected. The optical density was measured at 532 and 600 nm and the concentration of MDA-TBA concentration was calculated. The standard curve was produced by making a serial dilution of MDA stock solution to 1–10μM and treating the entire standard with TBA as described above [25].

### RNA sequencing, read data quality control, mapping and calculations of the differentially expressed genes

Total RNA was extracted from the roots of *V*. *angularis* plants in two groups: the control group and the 1.00 mg/L graphene group. Library construction and sequencing were carried out followingprevious reports [19, 26]. Three biological replicates were performed for each group. Raw data (raw reads) in fastq format were first processed through FASTX-Toolkit (http://hannonlab.cshl.edu/fastx_toolkit/) according to a previous study [26]. Clean data (clean reads) were obtained by removing low-quality reads, reads containing poly-N sequences, and reads containing adapters. Downstream analyses were performed based only on clean data. These clean reads were mapped to adzuki bean reference genome (https://www.ncbi.nlm.nih.gov/datasets/taxonomy/3914/) using HISAT2 software [27–30]. Only reads with a perfect match or one mismatch were further analyzed to calculate the expression values. Expression values were calculated using the fragments per kilobase of transcript per million fragments mapped (FPKM) method, which was determined by following the previous method from a prior study [26]. Differential expression analysis was conducted using DESeq2 [31]. The resulting *p* values were adjusted using Benjamini and Hochberg’s approach to control the false discovery rate (FDR). Genes with an adjusted *p* value of less than 0.01 and more than twofold expression change (up and down) were defined as differentially expressed. TBtools [32] was used to display the gene expression patterns from the FPKM values. The raw sequence data reported in this paper have been deposited in the Genome Sequence Archive in National Genomics Data Center [33, 34], China National Center for Bioinformation/Beijing Institute of Genomics, Chinese Academy of Sciences (GSA: accession number CRA011371) that are publicly accessible at https://ngdc.cncb.ac.cn/gsa.

### Gene annotation and enrichment analyses

Different databases were used to annotate the functions of differentially expressed genes in this study. These included the NCBI nonredundant protein and nucleotide sequence databases (ftp://ftp.ncbi.nih.gov/blast/db/), a manually annotated protein sequence database (http://www.uniprot.org/), Gene Ontology (GO, http://www.geneontology.org/), Clusters of Orthologous Groups of proteins (COG, http://www.ncbi.nlm.nih.gov/COG/), and Kyoto Encyclopedia of Genes and Genomes (KEGG, http://www.genome.jp/kegg/).

Gene Ontology (GO) enrichment analysis was conducted using the GOseq R package [35]. The statistical enrichment of differentially expressed genes in the KEGG pathways was tested using KOBAS [36]. Finally, the sequence of the DEG genes was aligned with NR, Swiss-Prot, COG, KOG, and KEGG databases using the DIAMOND software [37] with the following parameters: diamond -k 100 -e -evalue 1e-5 -f 5.

### Statistical analysis

For the analysis of variance (ANOVA) and Student’s t-test, we used the R package available at https://www.r-project.org/. To ensure that the data follows a Gaussian distribution, we conducted a normality test using the Shapiro-Wilk test. We used the least significant difference (LSD) test with a significance level of 5% to determine the significance of our results. All analyses were performed on at least three biological replicates for each sample.

## Results

### Characterization of graphene

**Fig 1:**
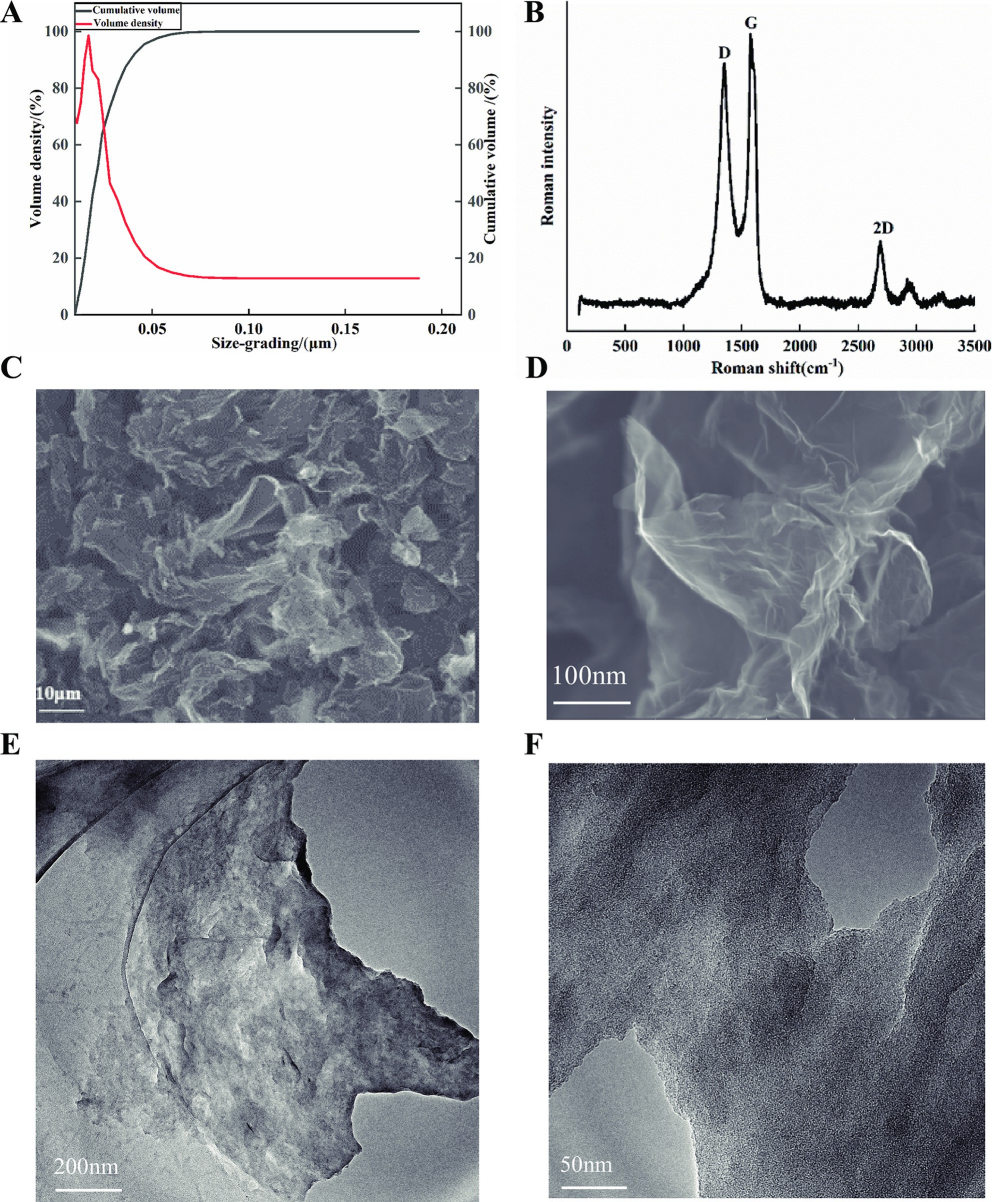
Characterization of graphene. (A) Sheets size distribution; (B) Raman spectra; (C, D) Scanning electron microscopy (SEM) image; (E, F) Transmission electron microscopy (TEM) image.

The size distribution of graphene particles showed that 10% of the particles were smaller than 13 nm, 50% were smaller than 21 nm, 90% were smaller than 38 nm, and the largest particle size observed was 88 nm (Fig 1A). The Raman spectrum (Fig 1B) revealed that the G peak was the primary characteristic peak of graphene, appearing at around 1575 cm^-1^, which was caused by the stretching vibration between sp^2^ carbon atoms. The D peak appeared near 1350 cm^-1^, which was caused by the symmetric stretching vibration of sp^3^ carbon atoms in the graphene sheets. The presence of the 2D peak, which appeared near 2690 cm^-1^, determined the number of graphene layers, and its shape was related to the number of layers of graphene [38, 39]. These results indicated that the graphene prepared in this study had a multi-layer structure. High-resolution scanning electron microscopy (Fig 1C and 1D) showed that the surface of graphene was folded, gauzy and disordered. Transmission electron microscopy revealed that graphene had a lamellar structure, stacked layer by layer (Fig 1E and 1F). The observation of a wide 2D peak in the Raman spectrum and TEM results indicated that the number of graphene layers prepared was within 5 layers.

### Effects of graphene on the seed emergence rate, plant height, stem diameter and leaf area of *Vigna angularis*

**Fig 2:**
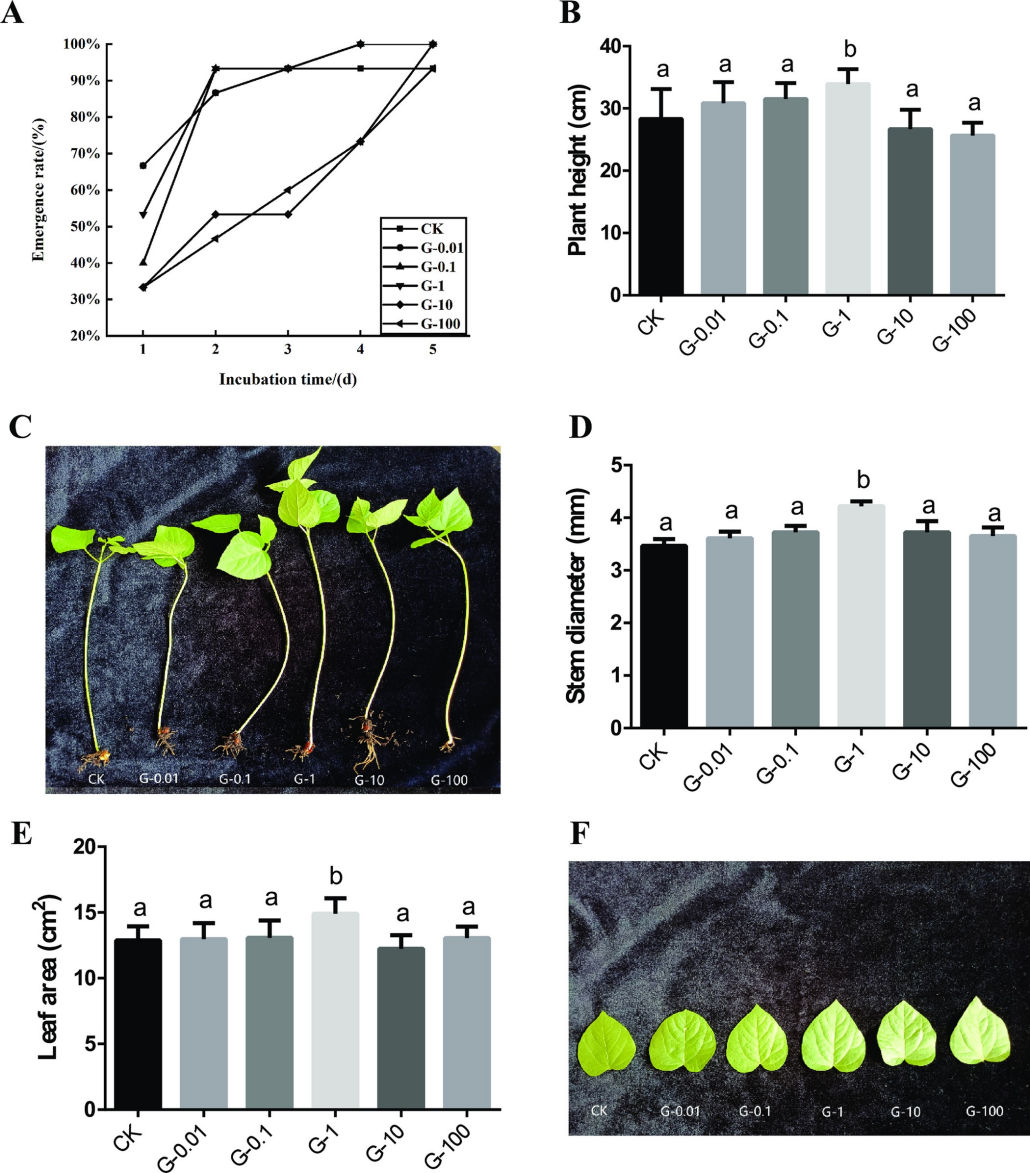
Effects of different concentrations of graphene on the emergence rate (A), plant height (B, C), stem diameter (D) and leaf area (E, F) of *V*. *angularis*. Note: Statistically significant differences (α = 0.05 level) of values are indicated with different letters with analysis of variance in R (https://www.r-project.org/).

To investigate the impact of graphene on the growth of *V*. *angularis* under soil culture conditions, the seed emergence rate, plant height, stem diameter and leaf area of *V*. *angularis* were measured (Fig 2). The emergence rate of *V*. *angularis* was calculated for five consecutive days after one week of planting with six concentrations of graphene: 0 mg/L (CK), 0.01 mg/L (G-0.01), 0.10 mg/L (G-0.1), 1.00 mg/L (G-1), 10.00 mg/L (G-10), 100.00 mg/L (G-100). Our results showed that graphene delayed the emergence time of *V*. *angularis* from the first to fourth days, except for the 1.00 mg/L group (G-1), which had an increased emergence rate (Fig 2A). On the fifth day, the emergence rate of all experimental groups was 100%, except for the CK group and G-10 group. These findings suggested that an appropriate concentration of graphene could improve the emergence rate of *V*. *angularis*. Furthermore, the plant height of the G-1 group increased significantly by 19.86% compared to the CK group, while there was no significant difference in the other experimental groups (Fig 2B and 2C). When the concentration of graphene was 1.00 mg/L, the stem diameter and leaf area of *V*. *angularis* reached a maximum of 4.19 mm and 15.56 cm^2^, respectively. This was a significant increase of 24.33% and 13.69% compared to the CK group (Fig 2D–2F). Overall, our results demonstrated that the effects of graphene on plant height, stem diameter, and leaf area of *V*. *angularis* were consistent. We found that a concentration of 1.00 mg/L graphene had the best promotion effects on *V*. *angularis* plant growth (Fig 2).

### Effects of graphene on the root architectures of *V*. *angularis*

**Fig 3:**
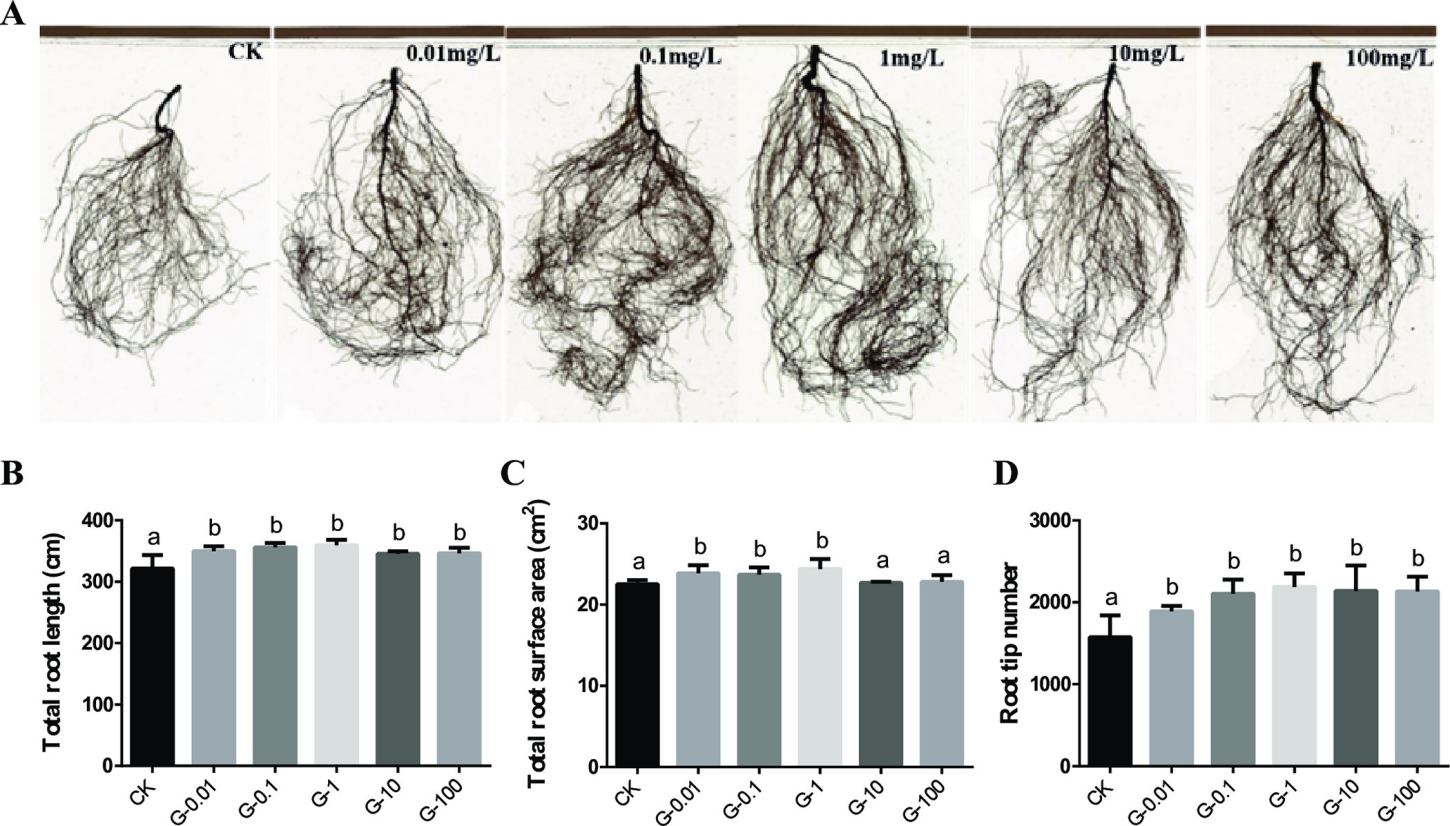
Effects of different concentrations of graphene on the root morphology of *V*. *angularis*. (A) root morphology; (B) total root length; (C) total root surface area and (D) root tip number. Note: Statistically significant differences (α = 0.05 level) of values are indicated with different letters with analysis of variance in R (https://www.r-project.org/).

The root morphology of *V*. *angularis* was studied under different concentrations of graphene in soil culture conditions, and the results were shown in Fig 3. It was observed that the roots of *V*. *angularis* were more developed after graphene treatment as compared to the control or CK group (Fig 3A). The total root length, total root surface area and the number of root tips of *V*. *angularis* also increased significantly after graphene treatment (Fig 3B–3D). The highest promotion effect of graphene was observed in the G-1 group, followed by G-0.1, G-0.01, G-100 and G-10 groups, respectively, as compared to the CK group (Fig 3B). The total root surface area of G-0.01, G-0.1 and G-1 groups increased significantly by 5.95%, 4.44% and 8.12%, respectively, as compared to the CK group (Fig 3C). As the concentration of graphene increased, the number of root tips also increased by 20.19%, 33.81%, 43.50%, 35.85% and 35.52%, respectively, as compared to the CK group (Fig 3D). Overall, the results suggested that all the experimental groups with graphene promoted the root growth and development of *V*. *angularis* plants, with the G-1 group showing the best promotion effect at a concentration of 1.00 mg/L.

### Effects of graphene on the leaf water potential, membrane permeability, malondialdehyde (MDA) content, and antioxidant enzyme activity of *V*. *angularis* plants

**Fig 4:**
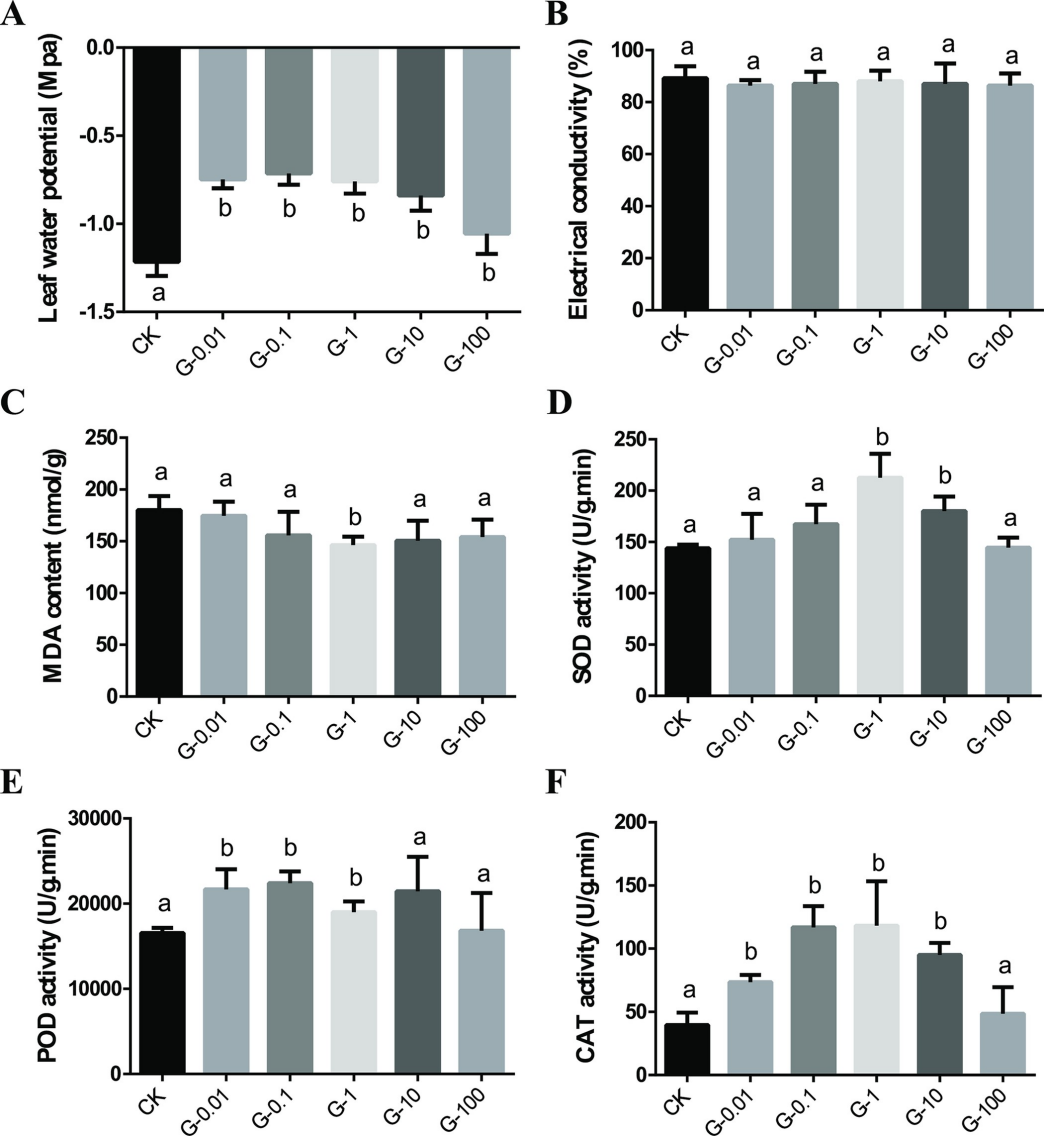
Effects of different concentrations of graphene on the leaf water potential (A), electrical conductivity (B), malondialdehyde content (C) and antioxidant enzyme SOD (D), POD (E) and CAT (F) activities of *V*. *angularis* plants. Statistically significant differences (α = 0.05 level) of values are indicated with different letters with analysis of variance in R (https://www.r-project.org/).

The effect of different concentrations of graphene on leaf water potential of *V*. *angularis* plants was determined in this study. The results showed that all graphene treatment groups had significantly higher leaf water potential compared to the CK group, with increases ranging from 13.11% to 42.62% (Fig 4A). However, the leaf conductivity in each graphene group slightly decreased by 1.12% to 3.37%, but these differences were not significant (Fig 4B). Moreover, the graphene group showed decreased malondialdehyde (MDA) content compared to the CK group, with the trend resembling a U-shaped curve (Fig 4C). We discovered that the MDA content was lowest when the graphene concentration was 1.00 mg/L. At this concentration, it was significantly reduced by 22.71% (Fig 4C). These results indicated that 1.00 mg/L graphene can help reduce the degree of plasma membrane damage, increase the leaf water potential and further to improve the stress resistance of *V*. *angularis* plants.

To investigate the impact of different concentrations of graphene solution on the antioxidant oxidase activities of *V*. *angularis* leaves, the activities of superoxide dismutase (SOD), peroxidase (POD) and catalase (CAT) in *V*. *angularis* leaves were measured after 30 days of soil cultivation. The results showed that the activities of SOD, POD and CAT increased initially and then decreased with the increase in graphene concentration (Fig 4D–4F). Compared to the CK group, SOD and CAT activities were highest at a graphene concentration of 1.00 mg/L. SOD activity in G-1 (1.00 mg/L) and G-10 (10.00 mg/L) groups increased significantly by 47.67% and 16.24%, respectively (Fig 4D). The POD activity treated with 0.01, 0.10 and 1.00 mg/L graphene solution increased significantly by 30.85%, 35.22% and 14.76%, respectively (Fig 4E). The CAT activity in G-0.1 (0.10 mg/L) and G-1 (1.00 mg/L) groups increased significantly by 195.7% and 199.3%, respectively (Fig 4F). These results indicated that increasing concentrations of graphene significantly enhanced the activities of SOD, POD, and CAT in *V*. *angularis* leaves to varying degrees, with the G-1 group showing higher levels. Our data indicated that there existed oxidative stress in the leaves of *V*. *angularis* plants exposed to different concentrations of graphene.

### Effects of graphene on the leaf photosynthetic properties, chlorophyll content, soluble sugar content of *V*. *angularis* plants

**Fig 5:**
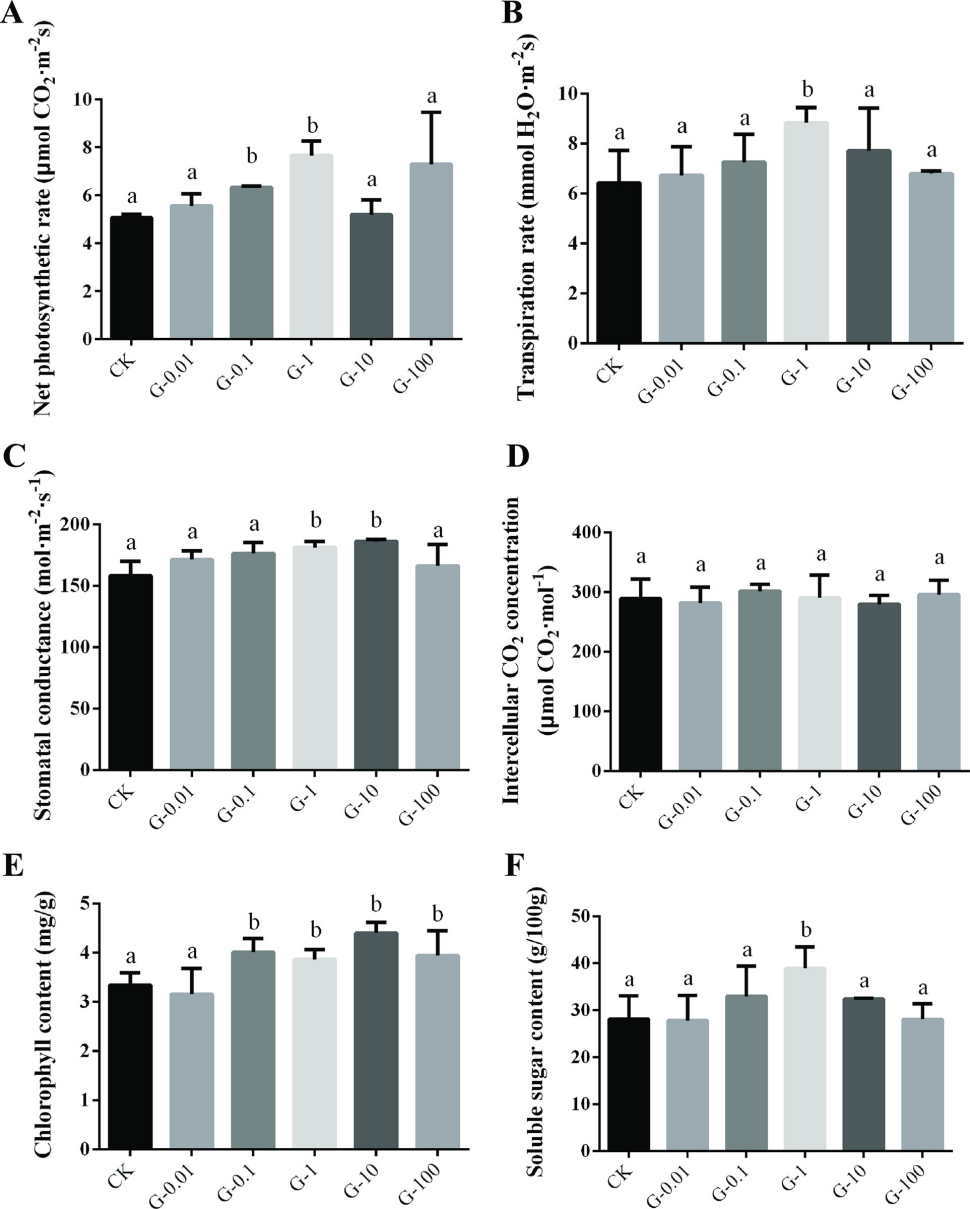
Effects of different concentrations of graphene on the leaf photosynthetic properties, chlorophyll content and soluble sugar content of *V*. *angularis* plants. (A) Net photosynthetic rate; (B) Transpiration rate; (C) Stomatal conductance; (D) Intercellular CO_2_ concentration; (E) Chlorophyll content; (F) Soluble sugar content. Note: Statistically significant differences (α = 0.05 level) of values are indicated with different letters with analysis of variance in R (https://www.r-project.org/).

Subsequently, the net photosynthetic rate, transpiration rate, and stomatal conductance of all the graphene treatment groups increased to varying degrees compared with that of the CK group (Fig 5A–5C). However, the intercellular CO_2_ concentration showed no significant difference between the treated and untreated groups (Fig 5D). Among them, the 1.00 mg/L graphene (G-1 group) had the most significant effect on promoting the leaf photosynthetic properties of *V*. *angularis* plants. Compared to the CK group, the net photosynthetic rate (Fig 5A), transpiration rate (Fig 5B) and stomatal conductance (Fig 5C) in G-1 group were increased by 51.28% (p<0.05), 37.54% (p<0.05) and 14.32% (p<0.05), respectively. Additionally, the chlorophyll contents of graphene treatment groups were increased to varying degrees, except for the G-0.01 group (Fig 5E). Compared to the CK group, the chlorophyll content of *V*. *angularis* leaves in the treated groups was increased by 20.06%, 24.25%, 24.1%, 17.96%, respectively (Fig 5E). Furthermore, the soluble sugar content in *V*. *angularis* leaves were significantly increased by 38.35% when graphene concentration was 1.00 mg/L (G-1 group) (Fig 5F). These results suggested that graphene can enhance photosynthesis and respiration, leading to an increase in chlorophyll and soluble sugar content in *V*. *angularis* plant leaves, thereby promoting plant growth and development.

### Effects of graphene on the podding rate, yield and rhizosphere soil physicochemical properties of *V*. *angularis* plants

**Fig 6:**
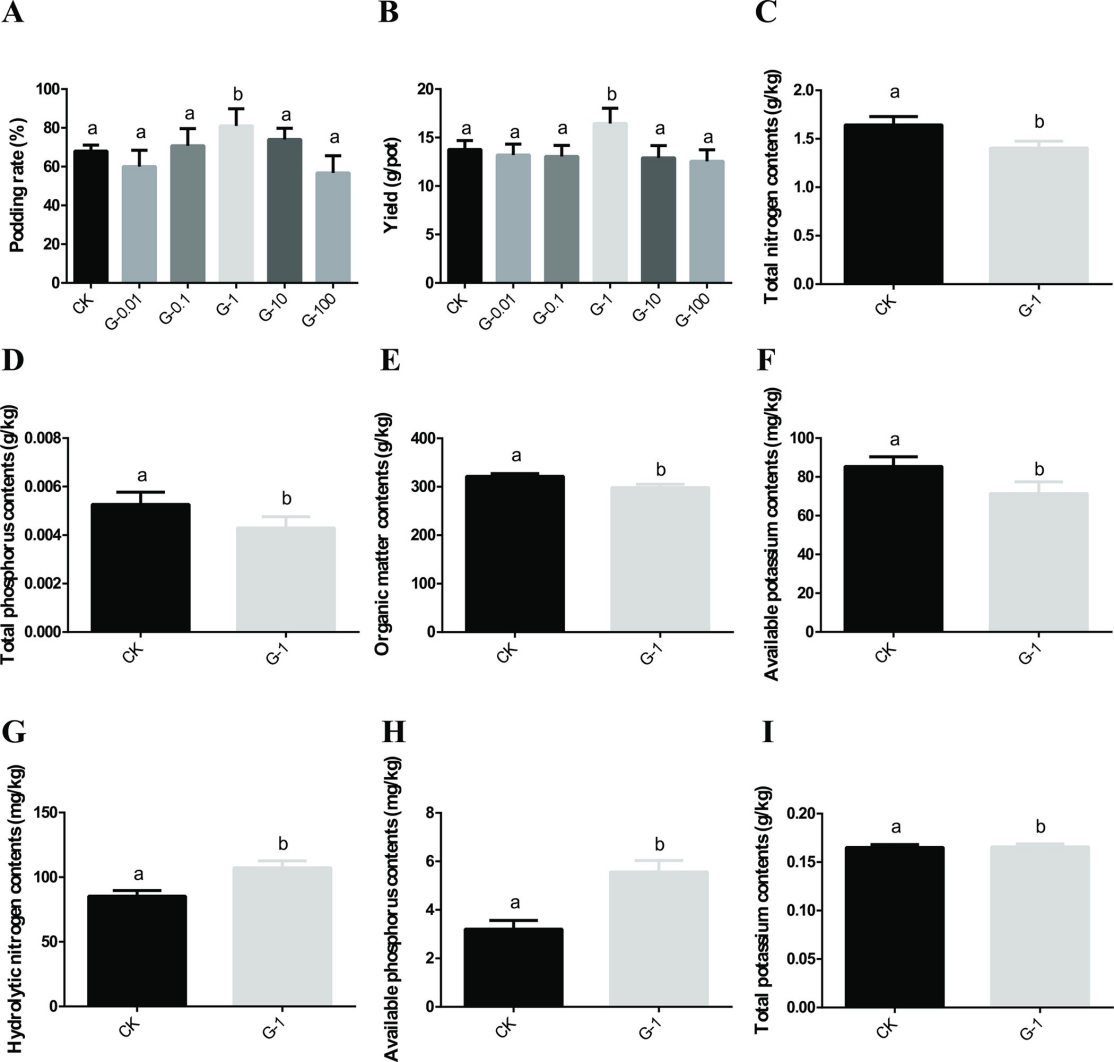
Effects of different concentrations of graphene on the podding rate, yield and physicochemical properties of *V*. *angularis* plants. (A) Podding rate; (B) Yield; (C) Total nitrogen contents; (D) Total phosphorus contents; (E) Organic matter content; (F) Available potassium contents; (G) Hydrolytic nitrogen contents; (H) Available phosphorus contents; (I) Total potassium contents. Note: Statistically significant differences (α = 0.05 level) of values are indicated with different letters with analysis of variance and Student’s t-test in R (https://www.r-project.org/).

To evaluate the effects of different concentrations of graphene solution on the production of *V*. *angularis* plants, the podding rate and yield of *V*. *angularis* plants were calculated. Fig 6 showed that graphene can significantly affect the pod rate and yield of *V*. *angularis* plants. When compared to the CK group, the podding rate and yield of *V*. *angularis* plants treated with 1.00 mg/L graphene showed an increase of 23.88% and 17.04%, respectively (Fig 6A and 6B). In summary, our results showed that 1.00 mg/L graphene was the most effective concentration for promoting the growth and development of *V*. *angularis* plants. To further understand the impact of graphene on *V*. *angularis* plants, the physicochemical properties of rhizosphere soil in CK and G-1 groups were measured. Notably, there was no significant difference in pH values between the G-1 and CK groups, which indicated that the soil solution remained neutral. However, when compared to the CK group, the soil of the G-1 group showed a decrease of 16.10% in total nitrogen (Fig 6C), 14.55% in total phosphorus (Fig 6D), 13.46% in organic matter (Fig 6E), and 5.23% in available potassium (Fig 6F). On the other hand, the contents of hydrolytic nitrogen (Fig 6G) and available phosphorus (Fig 6H) in the G-1 group increased by 25.14% and 66.67%, respectively. The total potassium content remained unchanged (Fig 6I). These results indicated that graphene could promote the degradation and utilization of total nitrogen, total phosphorus and organic matter, and increase the contents of hydrolyzed nitrogen and available phosphorus, ultimately leading to the growth, development and yield increase of *V*. *angularis* plants.

### Transcriptome sequencing and DEGs in *V*. *angularis* leaves after graphene treatment

To understand how graphene affects the growth and development of *V*. *angularis* plants at a molecular level, the leaf tissues were collected from *V*. *angularis* seedlings treated with 1.00 mg/L graphene (G-1) and untreated seedlings (CK) for transcriptome sequencing (S1 and S2 Tables). Pearson correlation coefficient (PCC) for all genes was calculated with all the correlation coefficients of the three biological replicates greater than 0.76 (S1A Fig). Principal component analysis (PCA) showed that 1.00 mg/L graphene and CK treatments exhibited two levels of gene expressions (S1B Fig). These results indicated that RNA-seq data were reliable for subsequent analysis. Using the fragments per kilobase of transcript per million fragments mapped (FPKM) algorithm, we calculated the expression level of each gene. A two-fold change and a *p*-value of less than 0.05 were set as the cutoffs to define genes with significant differential expression (S1C Fig). In total, 6,631 differentially expressed genes (DEGs) were identified, out of which 3,541 were up-regulated and 3,090 were down-regulated (S1D Fig).

### Gene enrichment analysis for DEGs

To investigate possible biological functions that determined the different responses of the *V*. *angularis* plants to 1.00 mg/L graphene treatment, GOseq [35] was used to perform GO category enrichment analysis for DEGs. S2 Fig lists the results of the GO analysis for DEGs after graphene treatment. GO terms associated with important biological processes were enriched after graphene treatment, including metabolic, cellular, single-organism, and developmental processes, biological regulation, response to stimulus, and detoxification. Additionally, cellular components, such as membrane, cell, cell part, membrane part, and organelle parts were enriched. Lastly, molecular function enrichment consisted of binding, catalytic activity, transporter activity, nucleic acid-binding transcription factor activity, antioxidant activity, and transcription factor activity.

The differentially expressed genes (DEGs) were analyzed using the COG database [40] to classify gene function and homology. As shown in S3 Fig, most of the DEGs were grouped into categories such as carbohydrate transport and metabolism, signal transduction mechanisms, secondary metabolites biosynthesis, transport and catabolism, lipid transport and metabolism, posttranslational modification, protein turnover, chaperones, amino acid transport and metabolism, as well as defense mechanisms.

**Fig 7:**
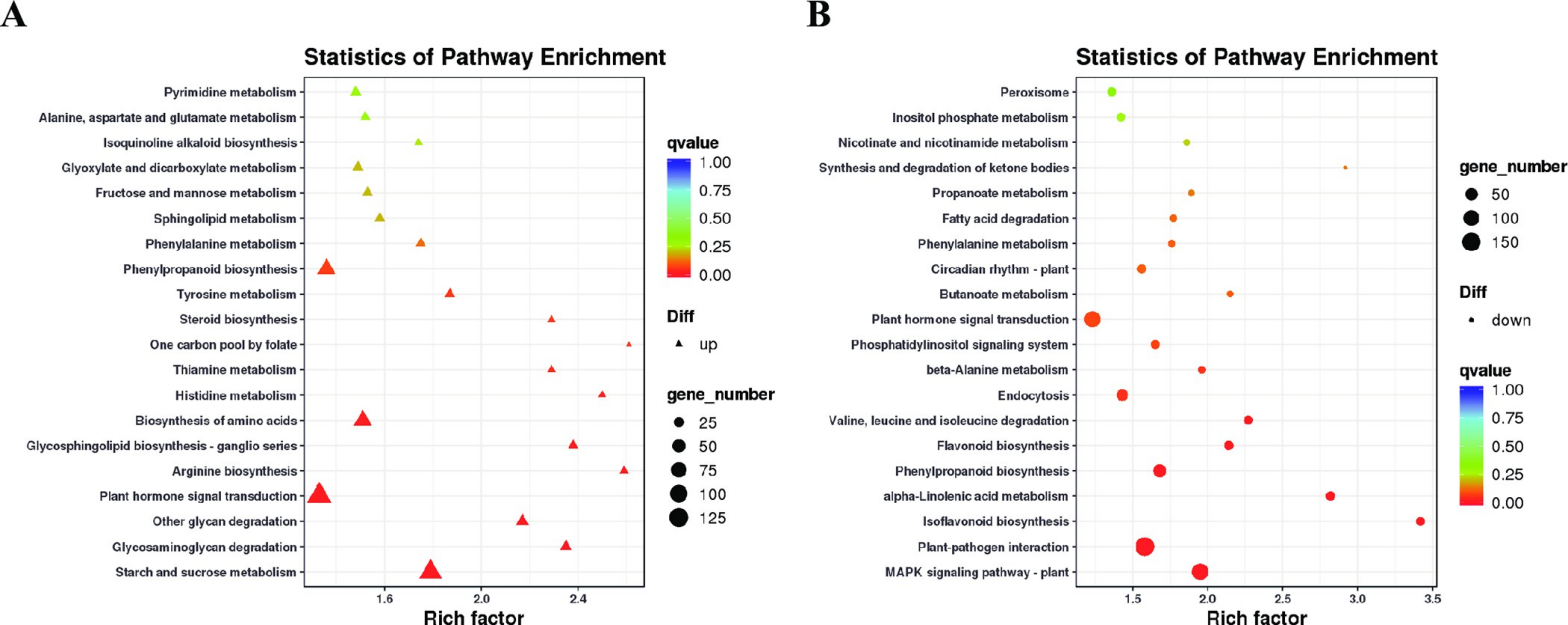
KEGG pathway analysis of enriched differentially expressed genes. (A) Top 20 pathways of significantly upregulated enriched genes (left). (B) Top 20 pathways of significantly downregulated (right) enriched genes.

DEGs were subjected to KEGG pathway analysis to identify the functional categorization. S4 Fig lists the results of the KEGG analysis for DEGs. Most DEGs were categorized belonging to the functional pathways related to: 1) metabolism, including phenylpropanoid biosynthesis, starch and sucrose metabolism, biosynthesis of amino acids, carbon metabolism, amino sugar and nucleotide sugar metabolism; 2) cellular process of endocytosis and peroxisomes; and 3) environmental information processing, including plant hormone signal transduction, ABC transporters, and phosphatidylinositol signaling system. The DEGs with upregulation of expression were assigned to 129 KEGG pathways, such as plant hormone signal transduction, starch and sucrose metabolism, glycosaminoglycan degradation, phenylpropanoid biosynthesis, carbon metabolism and nitrogen metabolism (Fig 7A). On the other hand, DEGs with downregulated expression were significantly enriched in 123 KEGG pathways, including plant hormone signal transduction, phenylpropanoid biosynthesis, starch and sucrose metabolism, carbon metabolism, ABC transporters and isoflavonoid biosynthesis **(**Fig 7B). The results revealed that graphene could affect the expression of *V*. *angularis* leaf genes, thereby exhibiting upregulated expression of a majority of genes. The enrichment analysis illustrated that graphene treatment exerted extensive and distinct effects on the life processes in *V*. *angularis*.

### Nitrogen metabolism enriched in *V*. *angularis* plants exposed to graphene

**Fig 8:**
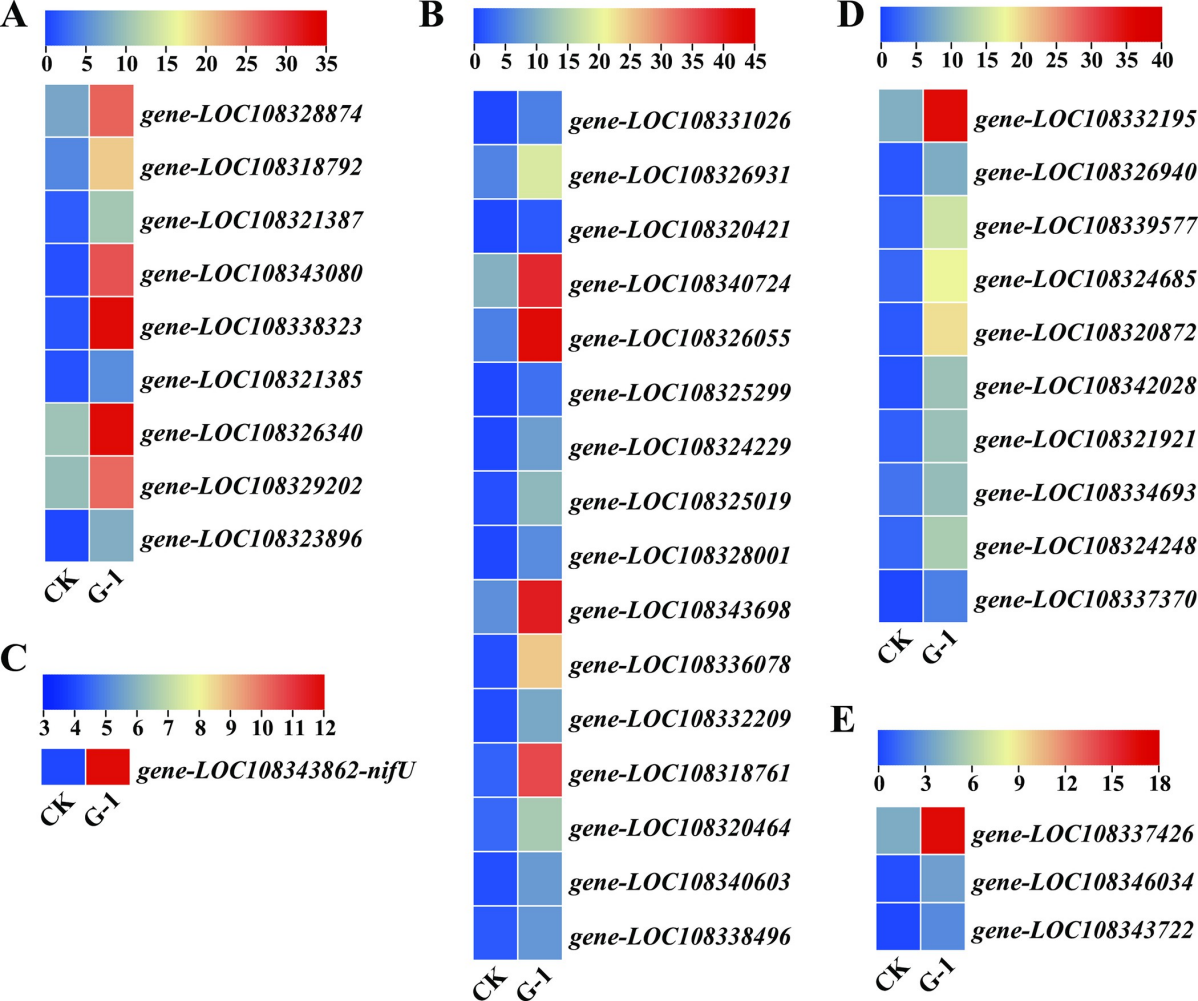
Upregulated expression of genes involved in nitrogen metabolism and photosynthesis genes. (A) Nine nitrogen metabolism gene expression profile based on RNA-seq data. (B) 16 NRT1/ PTR family genes gene expressions in CK and G-1 groups. (C) the expression of *NifU* nitrogenase gene in CK and G-1 groups. (D) ten photosynthesis genes expression profile in CK and G-1 groups. (E) three photosynthesis antenna proteins genes expression profile in CK and G-1 groups.

*V*. *angularis* belongs to the leguminous plant family and has specific nitrogen fixation function. Thus, we focused on the nitrogen metabolism pathway in this study, and the up-regulated DEGs were enriched in the nitrogen metabolism pathways. We totally identified nine nitrogen metabolism and 16 NRT1/ PTR family genes that were up-regulated expressed (Fig 8). All nine nitrogen metabolism genes were upregulated (Fig 8A). These genes were annotated in the glutamine synthetase, glutamate dehydrogenase 1, cyanate hydratase, and protein fluG-like (S3 Table), which were involved in the nitrogen transmembrane transport, uptake and root development. Additionally, the expression levels of 16 NRT1/ PTR family genes were upregulated based on RNA-seq (Fig 8B, S3 Table). NRT1/PTR FAMILY (NPF) proteins could transport not only nitrate, but also plant hormones such as auxin, abscisic acid, and gibberellin, as well as secondary metabolites (glucosinolates) [41]. In our study, the expression of *NifU* nitrogenase gene was up-regulated (Fig 8C, S3 Table). Consequently, these results indicated that graphene affected the nitrogen metabolism pathway to promote the growth and development of *V*. *angularis* plants.

### Graphene enhances the expression of photosynthesis metabolism genes

Since this study found that graphene can significantly promote root development, increase biomass and yield, we then focused on the effect of graphene on the photosynthesis of *V*. *angularis* plants. As previously mentioned, graphene elevated the leaf photosynthetic properties, chlorophyll content, soluble sugar content of the plants (Fig 5). Here, our analysis revealed ten up-regulated photosynthesis genes (annotated in the ATP synthase subunit b, photosystem I reaction center subunit XI, photosystem I reaction center subunit IV A, ferredoxin and psbP-like protein 1) that were involved in the photosynthesis process (as shown in Fig 8D and S3 Table). Furthermore, three photosynthesis antenna proteins genes were also found to be upregulated based on RNA-seq data (Fig 8E, S3 Table). Combining physiological and RNA-seq data, our results indicated that graphene enhanced the photosynthesis process, which in turn promoted the growth and development of *V*. *angularis* plants.

## Discussions

In this study, the effects of graphene on the growth and development of *V*. *angularis* plants were investigated through soil culture experiments, and six concentrations were selected to study the impact of graphene on physiological characteristics and gene expressions of *V*. *angularis*, which providing a research basis for the application of graphene in agricultural production. Malondialdehyde (MDA), a substance produced by membrane lipids in response to reactive oxygen species (ROS), can act as an adverse stress indicator to evaluate the degree of plasma membrane damage and the ability of plants to stress tolerance [42]. Antioxidant enzyme systems could reduce the oxidative stress injury suffered by plants in response to various stresses [21]. Some studies have shown that graphene was beneficial to the better absorption of water in plant seeds, alleviate the oxidative stress and promote the germination of plant seeds [13, 15], which was consistent with the results in this study. One study even found that graphene promoted the stem elongation and increased plant height by about 18% in tomato [13].

Studies have shown that treating plants with low doses of graphene improved their root growth and development. For example, low doses of graphene have been found to promote the root growth of several plant species, including *Rubus corchorifolius* [43, 44], *Chenopodium quinoa* [45], and rice plants [46]. Furthermore, low concentration of graphene have been shown to increase the total root length and root volume of maize plants [26], increase the number of root tips and hairs in tomato plants [47] and improve the morphological characteristics of roots in *Aloe vera* [48]. These results can be explained by the fact that a certain concentration of graphene could increase the contact area between the roots and the nutrients in the soil, thus promoting the absorption of nutrients by the roots, and further to promote root growth. Total root length and surface area were the most commonly used parameter for studies of root growth in response to the environment [49]. Root numbers generally provided a good impression of the rooting density in a soil profile. Thus, higher total root length and surface area in seedlings were subjected to nitrogen and phosphorus deprivation to increase the plant biomass [50].

Plant water potential can directly reflect the degree of water shortage and drought resistance of plants, as well as the ability of plants to obtain water from the soil [51]. Among the water potential of different plant organs, such as the root, stem, and leaf, the leaf water potential is the most sensitive to water deficit of plants [52]. Researchers have found that graphene could act as an effective soil water retention agent and confer drought stress tolerance to *Paeonia ostia* [53]. Graphene helps to retain soil water and reduce the loss of soil water. Furthermore, Yin et al. [54] showed that under metal stress of Cd^2+^, the growth of maize seedlings slowed down, but low concentration of graphene could relieve the Cd^2+^stress. Pandey et al. [55] found that under salt stress, long-term application of graphene could increase the number of flowers and fiber biomass of cotton. In our study, the available water of plants increased and the water stress was weakened, thus the leaf water potential of *V*. *angularis* plants increased. These results indicated that an appropriate concentration of graphene can improve the leaf water potential and alleviate drought stress in *V*. *angularis* plants.

In addition, in the process of plant growth and development, photosynthesis and transpiration are two indispensable processes, but there is a significant correlation between plant transpiration rate and net photosynthetic rate [56]. To a certain extent, the net photosynthetic rate and transpiration rate of plants can directly reflect the intensity of photosynthesis, and the photosynthesis of plants is mainly conducted through the exchange of CO_2_ and water through leaf stomata [57, 58]. Stomatal conductance directly affects photosynthesis and photosynthetic rate of plants [57]. Our results of this study showed that using an appropriate concentration of graphene (e.g. 1.00 mg/L) could significantly improve the net photosynthetic rate, transpiration rate and stomatal conductance of *V*. *angularis* plants. The previous study investigated the physiological responses of graphene on *Aloe vera*. Their results demonstrated that 50 mg/L graphene could enhance the photosynthetic capacity of leaves, increase the biomass of root and leaf, and improve the protein and amino acid contents of leaf [48]. In addition, graphene could alleviate salinity and alkalinity stresses in alfalfa by enhancing its leaf photosynthesis through regulating their gene expression [16]. Another study found that 25 mg/L of graphene could significantly promote plant root growth by enhancing root respiration [19]. Chlorophyll content directly affects the photosynthesis of plants, which further affects the photosynthetic efficiency of plants and ultimately affects the growth of plants [59]. The content of soluble sugar directly reflects the carbon nutrition status from photosynthesis in plants [60]. Our results indicated that graphene treatment increased the chlorophyll content in *V*. *angularis* plant leaves, which further significantly enhanced the photosynthesis of *V*. *angularis* leaves, increased the efficiency of CO_2_ conversion into organic matter, and finally increased the soluble sugar content of leaves. These results suggested that graphene can enhance plant growth and development by improving photosynthesis and respiration.

Many researches have utilized RNA-seq technologies to gain new insights into the molecular mechanisms of graphene’s positive effects on plant growth and development. For instance, our previous research has revealed that the nitrogen and potassium metabolism genes were upregulated in maize roots after graphene treatment [26]. In *Pinus tabuliformis* roots, the proline synthesis genes were induced by graphene [61]. In tomato roots, graphene significantly increased the expression of *SlExt1* and *LeCTR1* root development genes [47]. Additionally, the lignin biosynthesis and photosynthesis-antenna protein genes were also induced by graphene treatment [53]. A recent study showed that graphene affected the expressions of glycolysis/gluconeogenesis, pyruvate metabolism, citrate cycle (TCA cycle) metabolism genes to promote the growth of plant roots [19].

Previous research has shown that graphene can enhance plant root growth and development by affecting carbon metabolism [19]. In the current study, we observed an increase in the leaf net photosynthetic rate, chlorophyll content, and soluble sugar content after graphene treatment compared to the CK group. In view of these observations, we focused on the differentially expressions of photosynthesis genes based on the RNA-seq data. In detail, the expression levels of ATP synthase subunit b, photosystem I reaction center subunit XI, photosystem I reaction center subunit IV A, ferredoxin and psbP-like protein 1, photosynthesis antenna proteins genes were induced after graphene treatment, indicating that the photosynthesis was enhanced in *V*. *angularis* plants. Similar results were also observed in a previous study where uptake of graphene enhanced the photophosphorylation process in rice plants [62]. Therefore, it can be concluded that graphene does affect the photosynthesis process.

Our previous results showed that 50.00 mg/L graphene could significantly increase the content of available nitrogen contents in *Zea mays* rhizosphere soil [26]. Here, graphene increased the content of hydrolyzable nitrogen in *V*. *angularis* rhizosphere soil. RNA-seq data showed that DEGs were also involved in the glutamine synthetase, glutamate dehydrogenase 1, cyanate hydratase, protein fluG-like, NRT1/PTR family genes and *NifU* nitrogenase gene. Study showed that graphene oxide promoted the number of root nodules per plant of mungbean plant [18]. Actually, *V*. *angularis* plants can convert atmospheric dinitrogen to ammonium by biological nitrogen fixation [63]. The conventional nitrogenase enzyme was encoded by the *nifHDK* genes, however the NifU and NifS proteins were involved in the production of active forms of the nitrogenase component proteins, NifH and NifDK [64, 65], which suggesting that graphene promoted the growth and development of *V*. *angularis* by enhancing the nitrogen metabolism processes.

As graphene applications expanded in agriculture, it might cause potential environmental safety issues in long term. Current study showed that the polycyclic structure of graphene is similar to that of lignin, and further study indicated that graphene can be degraded by lignin peroxidase enzymes secreted by fungi in soil [66]. In addition, some bacteria could utilize graphene as a carbon source to support their growth [67]. Other researchers have demonstrated that when ^14^C-labeled graphene was introduced to rice leaves, it reacted with OH. This led to the degradation of graphene into ^14^CO2, with no graphene remaining in rice seeds [68]. These findings are reassuring in terms of public safety concerns about the long-term impacts of graphene on crop production, given its degradability.

The impact of graphene and its related materials on plants is closely related to various factors such as the dose, size, structure, treatment time, solubility, plant species and growth conditions. As the application of graphene becomes more and more widespread, including applying graphene to improve the utilization efficiency of water, fertilizers and insecticides [69, 70], the impact of graphene on biology has also attracted more and more attention. However, the effect and the mechanism of action of graphene on plants needs still to be studied.

## Conclusions

In this study, we investigated the effects of different concentrations of graphene on the growth and physiological characteristics of *V*. *angularis* plants in soil, and various indexes were measured comprehensively. The results showed that within the tested concentration range, graphene had no inhibitory effect on the growth of *V*. *angularis*. In fact, low concentrations of graphene promoted the growth of *V*. *angularis* by increasing plant height, stem diameter, leaf area, and root system development. Among all tested concentrations, 1.00 mg/L graphene had the most significant effect. It resulted in increased accumulation of soluble sugar and chlorophyll content, higher activity of antioxidant enzymes, decreased content of malondialdehyde, and higher yield of *V*. *angularis* plants. The transcriptome results showed that 1.00 mg/L of graphene induced the upregulation of 3,590 genes and inhibited the expression of 3,090 genes. KEGG annotation results showed that DEGs were significantly enriched in starch—sucrose metabolism and nitrogen metabolism pathways, suggesting that graphene could affect the growth and development of *V*. *angularis* plants by regulating the photosynthesis and biological nitrogen fixation processes. In conclusion, the appropriate concentration of graphene can positively impact the growth and development of *V*. *angularis* plants in soil. Our result provided a certain reference value for the application of graphene in agriculture.

## Supporting information

S1 Fig Overview of *V*. *angularis* leaf transcriptome response to 1.00 mg/L graphene and the control (CK). (A) Pearson correlation coefficient (PCC) analysis of all genes between the six samples. (B) Principal component analysis (PCA) of all samples. Red and light blue colors represent the samples of CK and those exposed to 1.00 mg/L graphene, respectively. (C) Volcano plot of differentially expressed genes. (D) The number of upregulated and downregulated genes.
(TIF)

S2 Fig Gene ontology (GO) enrichment analysis of differentially expressed genes (DEGs) after exposure to graphene. The X-axis represents the biological functions (molecular function, biological process, and cellular component) of these DEGs. The Y-axis represents the percentage or number of genes categorized into different functional pathways.
(TIF)

S3 Fig COG database for classification of the differentially expressed gene function and homology. (TIF)

S4 Fig KEGG pathway analysis for identification of the differentially expressed gene functional categorization. (TIF)

S1 Table Characteristics of the RNA-sequencing data from six root samples of *V*. *angularis*. (DOCX)

S2 Table The mapping results of RNA-seq clean reads from six root samples using the *V*. *angularis* genome. (DOCX)

S3 Table Five metabolism pathway genes induced by graphene and their NR_annotation information. (DOCX)

S1 Dataset (XLSX)

## List of abbreviations

DEGs: Differentially expressed genes
GO: Gene ontology
COG: Clusters of Orthologous Groups
FPKM: Fragments per kilobase of transcript per million mapped fragments
POD: Peroxidase
SOD: Superoxide dismutase
CAT: Catalase
PCA: Principal component analysis
ns: not significant
SEM: Scanning electron microscopy
TEM: Transmission electron microscopy
