# Supplementary figures and images for: Graphene promotes the growth of Vigna angularis by regulating the nitrogen metabolism and photosynthesis
Source: PLoS One. 2024 Mar 7;19(3):e0297892. doi: 10.1371/journal.pone.0297892 (PMC10919591; doi:10.1371/journal.pone.0297892)

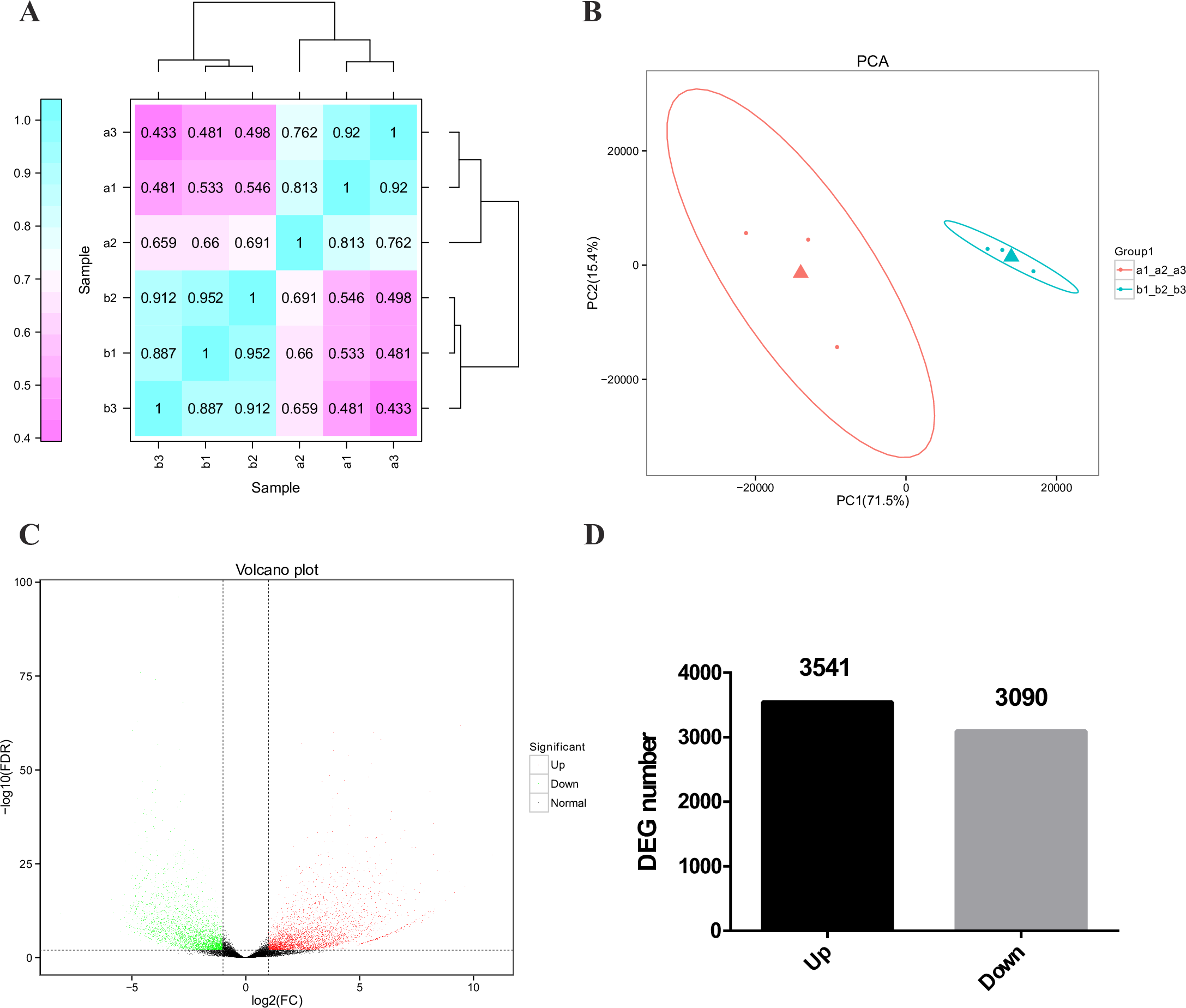

Supplement: S1 Fig — (A) Pearson correlation coefficient (PCC) analysis of all genes between the six samples. (B) Principal component analysis (PCA) of all samples. Red and light blue colors represent the samples of CK and those exposed to 1.00 mg/L graphene, respectively. (C) Volcano plot of differentially expressed genes. (D) The number of upregulated and downregulated genes. (TIF) [file pone.0297892.s001.tif]

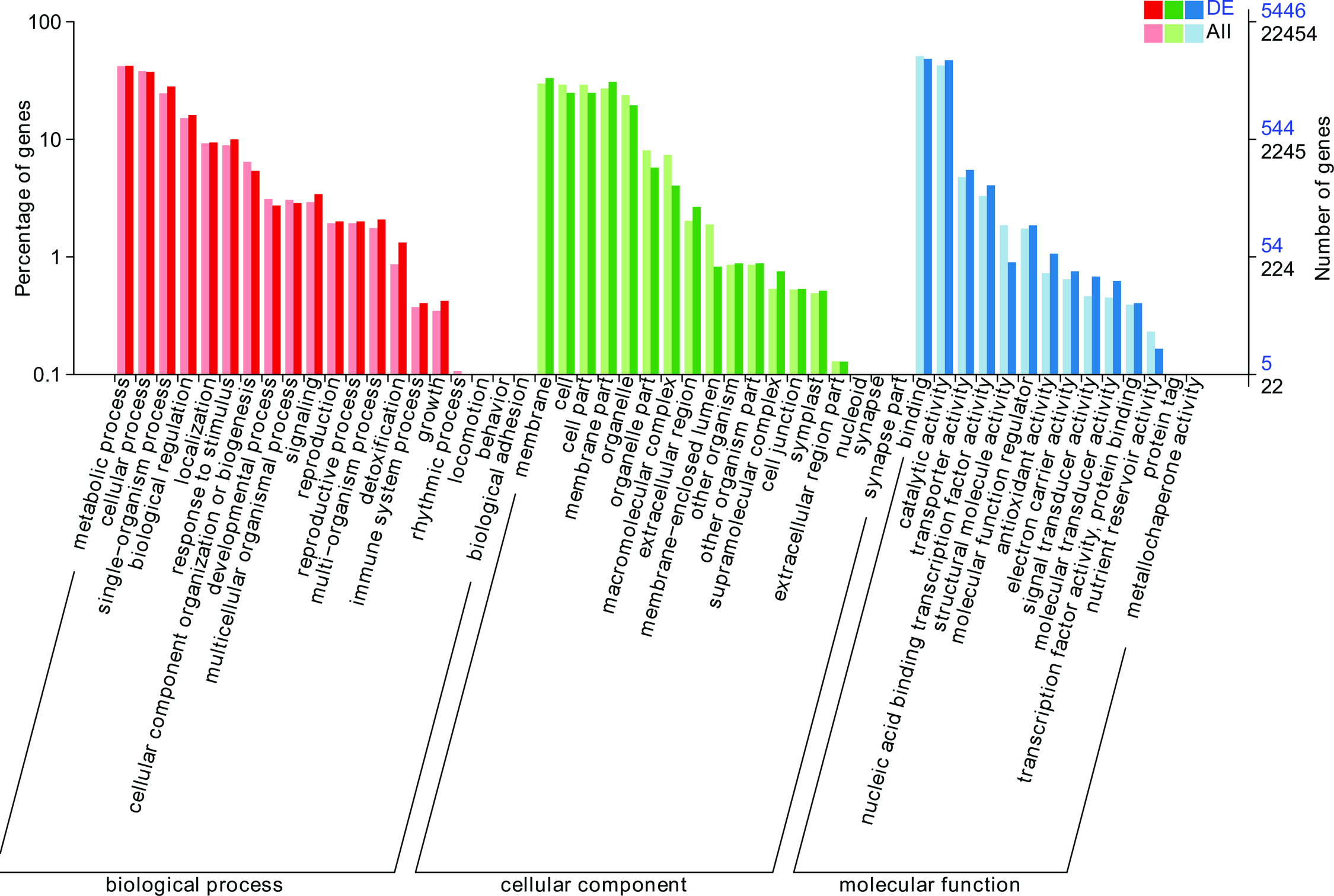

Supplement: S2 Fig — The X-axis represents the biological functions (molecular function, biological process, and cellular component) of these DEGs. The Y-axis represents the percentage or number of genes categorized into different functional pathways. (TIF) [file pone.0297892.s002.tif]

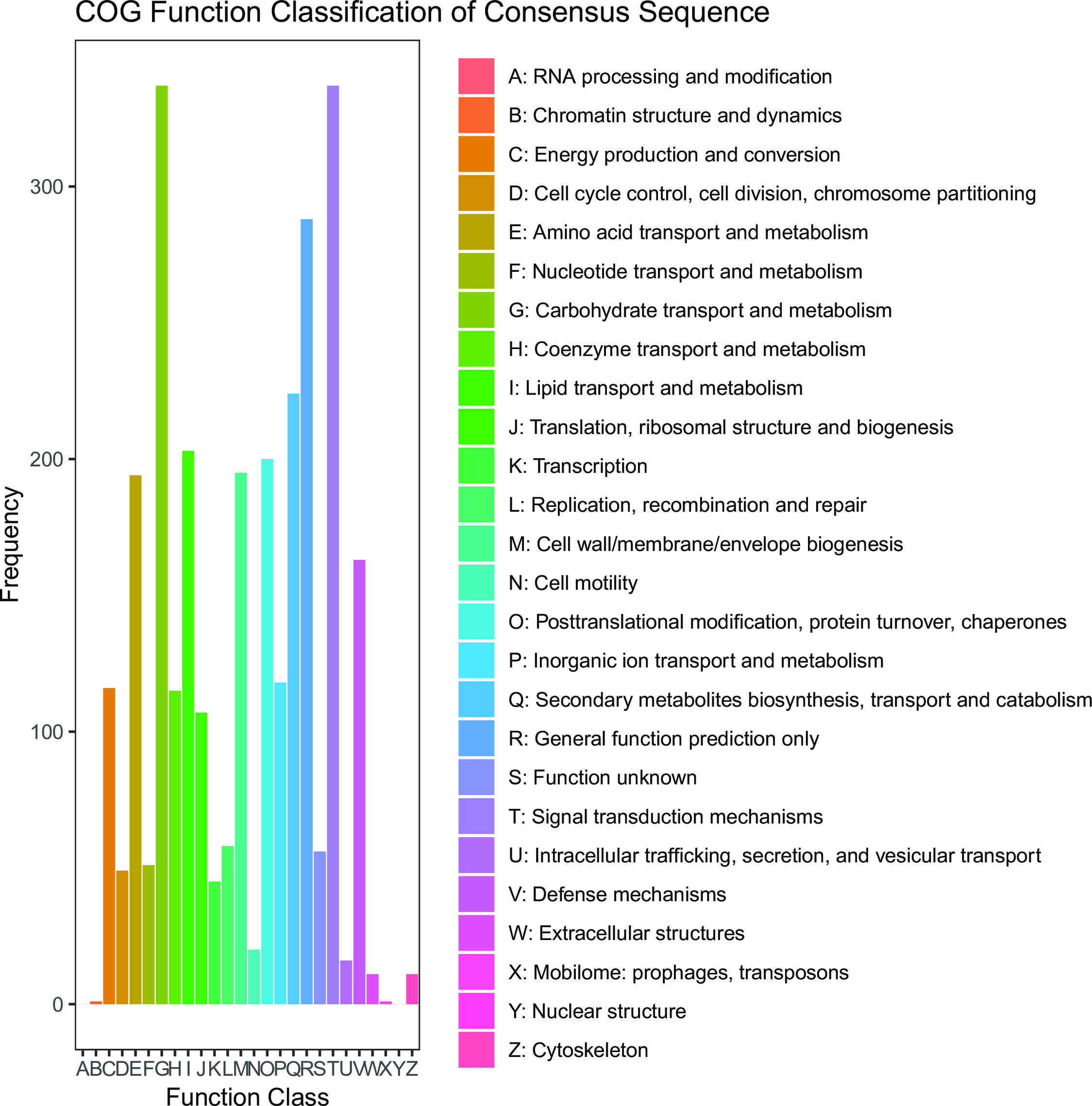

Supplement: S3 Fig — (TIF) [file pone.0297892.s003.tif]

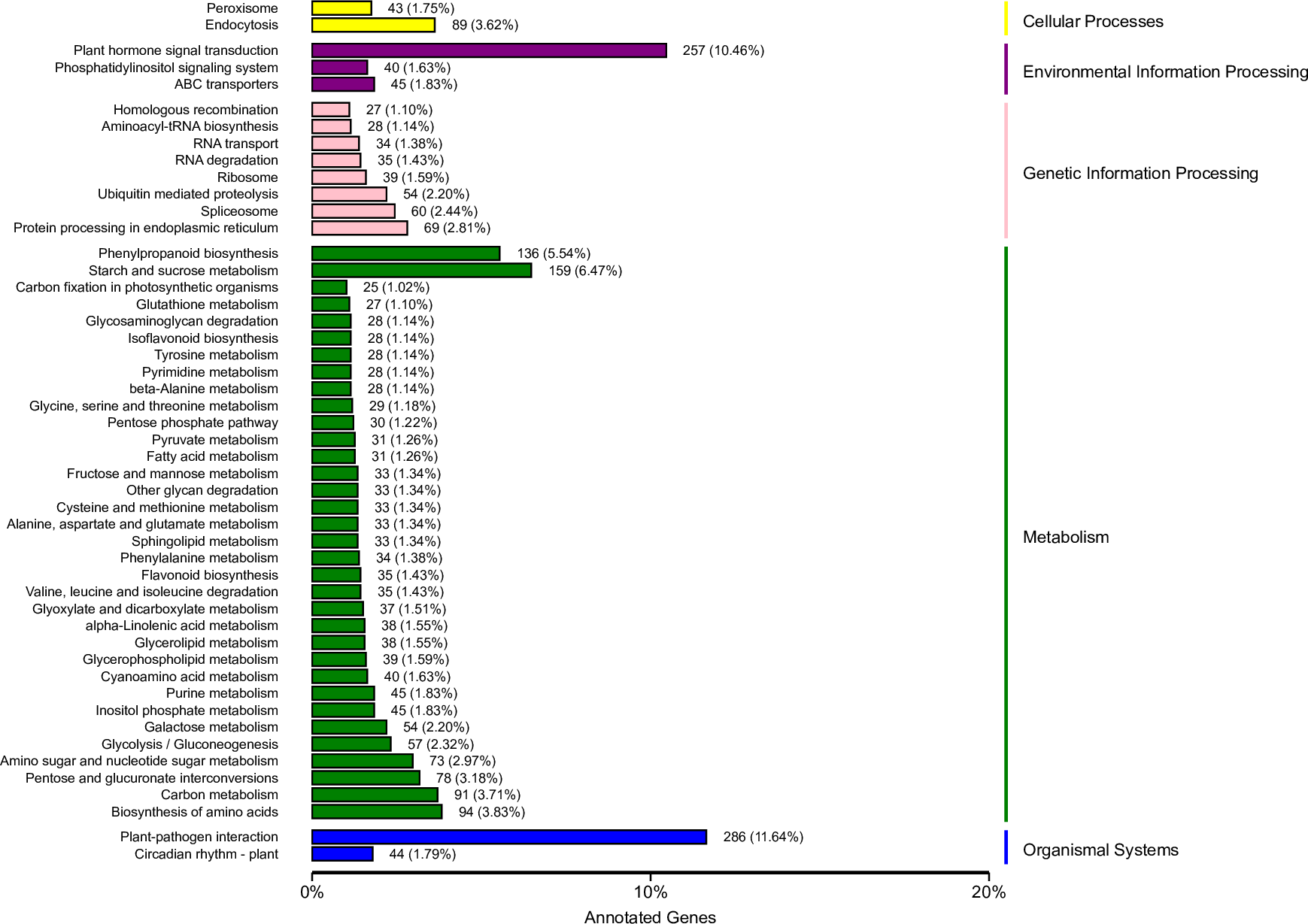

Supplement: S4 Fig — (TIF) [file pone.0297892.s004.tif]
