# Supplementary material for: Graphene promotes the growth of Vigna angularis by regulating the nitrogen metabolism and photosynthesis
Source: PLoS One. 2024 Mar 7;19(3):e0297892. doi: 10.1371/journal.pone.0297892 (PMC10919591; doi:10.1371/journal.pone.0297892)
Supplement: S1 Table — (DOCX) [file pone.0297892.s005.docx]

Table S1. Characteristics of the RNA-sequencing data from six root samples of *V. angularis*.

| Samples | Clean reads | Clean bases | GC Content | %≥Q30 |
| --- | --- | --- | --- | --- |
| CK-1 | 24,753,624 | 7,409,437,398 | 52.12% | 94.68% |
| CK-2 | 25,809,975 | 7,723,459,548 | 49.82% | 94.22% |
| CK-3 | 21,003,018 | 6,285,721,062 | 51.16% | 94.43% |
| G-1-1 | 36,945,956 | 11,043,226,278 | 45.86% | 95.65% |
| G-1-2 | 35,918,929 | 10,745,156,826 | 46.55% | 94.72% |
| G-1-3 | 31,121,210 | 9,310,399,740 | 45.31% | 95.24% |

Note: G-1 represents root samples treated with 1.00 mg/L graphene.
