# Supplementary material for: Graphene promotes the growth of Vigna angularis by regulating the nitrogen metabolism and photosynthesis
Source: PLoS One. 2024 Mar 7;19(3):e0297892. doi: 10.1371/journal.pone.0297892 (PMC10919591; doi:10.1371/journal.pone.0297892)
Supplement: S2 Table — (DOCX) [file pone.0297892.s006.docx]

Table S2. The mapping results of RNA-seq clean reads from six root samples using the *V. angularis* genome.

| Sample ID | Total Reads | Mapped Reads | Unique Mapped Reads | Multiple Mapped Reads | Reads Map to “+” | Reads Map to “-” |
| --- | --- | --- | --- | --- | --- | --- |
| CK-1 | 49,507,248 | 10,896,307 (22.01%) | 10,513,334 (21.24%) | 382,973 (0.77%) | 5,709,376 (11.53%) | 5,723,542 (11.56%) |
| CK-2 | 51,619,950 | 25,775,154 (49.93%) | 24,837,455 (48.12%) | 937,699 (1.82%) | 13,545,202 (26.24%) | 13,561,386 (26.27%) |
| CK-3 | 42,006,036 | 8,903,747 (21.20%) | 8,619,059 (20.52%) | 284,688 (0.68%) | 4,648,981 (11.07%) | 4,658,038 (11.09%) |
| G-1-1 | 73,891,912 | 63,037,872 (85.31%) | 60,766,416 (82.24%) | 2,271,456 (3.07%) | 33,151,648 (44.87%) | 33,204,864 (44.94%) |
| G-1-2 | 71,837,858 | 54,952,386 (76.50%) | 53,023,253 (73.81%) | 1,929,133 (2.69%) | 28,828,908 (40.13%) | 28,901,967 (40.23%) |
| G-1-3 | 62,242,420 | 57,245,806 (91.97%) | 55,115,765 (88.55%) | 2,130,041 (3.42%) | 30,125,870 (48.40%) | 30,185,695 (48.50%) |

Note: G-1 represents root samples treated with 1.00 mg/L graphene.
