# Supplementary material for: Graphene promotes the growth of Vigna angularis by regulating the nitrogen metabolism and photosynthesis
Source: PLoS One. 2024 Mar 7;19(3):e0297892. doi: 10.1371/journal.pone.0297892 (PMC10919591; doi:10.1371/journal.pone.0297892)
Supplement: S3 Table — (DOCX) [file pone.0297892.s007.docx]

**Table S3.** Five metabolism pathway genes induced by graphene and their NR_annotation information.

| Metabolism pathway | **Gene ID** | **NR_annotation** |
| --- | --- | --- |
| Nitrogenase gene | gene-LOC108343862 | PREDICTED: nifU-like protein 3, chloroplastic [Vigna angularis] |
| NRT1/ PTR FAMILY | gene-LOC108331026 | PREDICTED: protein NRT1/ PTR FAMILY 8.2-like [Vigna angularis] |
|  | gene-LOC108326931 | PREDICTED: protein NRT1/ PTR FAMILY 4.6 [Vigna angularis] |
|  | gene-LOC108320421 | PREDICTED: protein NRT1/ PTR FAMILY 5.6-like [Vigna angularis] |
|  | gene-LOC108340724 | PREDICTED: protein NRT1/ PTR FAMILY 7.1-like [Vigna angularis] |
|  | gene-LOC108326055 | PREDICTED: protein NRT1/ PTR FAMILY 7.3-like [Vigna angularis] |
|  | gene-LOC108325299 | PREDICTED: protein NRT1/ PTR FAMILY 8.1 [Vigna angularis] |
|  | gene-LOC108324229 | PREDICTED: protein NRT1/ PTR FAMILY 1.2-like [Vigna angularis] |
|  | gene-LOC108325019 | PREDICTED: protein NRT1/ PTR FAMILY 5.2-like [Vigna angularis] |
|  | gene-LOC108328001 | PREDICTED: protein NRT1/ PTR FAMILY 7.1 [Vigna angularis] |
|  | gene-LOC108343698 | PREDICTED: protein NRT1/ PTR FAMILY 4.5-like [Vigna angularis] |
|  | gene-LOC108336078 | PREDICTED: protein NRT1/ PTR FAMILY 6.2 [Vigna angularis] |
|  | gene-LOC108332209 | PREDICTED: protein NRT1/ PTR FAMILY 2.13-like [Vigna angularis] |
|  | gene-LOC108318761 | PREDICTED: protein NRT1/ PTR FAMILY 3.1 [Vigna angularis] |
|  | gene-LOC108320464 | PREDICTED: protein NRT1/ PTR FAMILY 6.3-like [Vigna angularis] |
|  | gene-LOC108340603 | PREDICTED: protein NRT1/ PTR FAMILY 1.1-like [Vigna angularis] |
|  | gene-LOC108338496 | PREDICTED: protein NRT1/ PTR FAMILY 6.4 [Vigna angularis] |
| Nitrogen metabolism | gene-LOC108328874 | PREDICTED: ferredoxin-dependent glutamate synthase, chloroplastic isoform X1 [Vigna angularis] |
|  | gene-LOC108318792 | PREDICTED: type-1 glutamine synthetase 1-like, partial [Vigna angularis] |
|  | gene-LOC108321387 | PREDICTED: protein fluG-like [Vigna angularis] |
|  | gene-LOC108343080 | PREDICTED: glutamine synthetase leaf isozyme, chloroplastic [Vigna angularis] |
|  | gene-LOC108338323 | PREDICTED: glutamine synthetase N-1 [Vigna angularis] |
|  | gene-LOC108321385 | PREDICTED: protein fluG-like, partial [Vigna angularis] |
|  | gene-LOC108326340 | PREDICTED: cyanate hydratase [Vigna angularis] |
|  | gene-LOC108329202 | PREDICTED: glutamate dehydrogenase 1 [Vigna angularis] |
|  | gene-LOC108323896 | PREDICTED: formamidase-like isoform X1 [Vigna angularis] |
| Photosynthesis | gene-LOC108332195 | PREDICTED: ATP synthase subunit b', chloroplastic [Vigna angularis] |
|  | gene-LOC108326940 | PREDICTED: photosystem I reaction center subunit XI, chloroplastic [Vigna angularis] |
|  | gene-LOC108339577 | PREDICTED: photosystem I reaction center subunit IV A, chloroplastic-like [Vigna angularis] |
|  | gene-LOC108324685 | PREDICTED: ferredoxin-1 [Vigna angularis] |
|  | gene-LOC108320872 | PREDICTED: ferredoxin [Vigna angularis] |
|  | gene-LOC108342028 | PREDICTED: ferredoxin, leaf L-A-like [Vigna angularis] |
|  | gene-LOC108321921 | PREDICTED: ATP synthase gamma chain, chloroplastic-like [Vigna angularis] |
|  | gene-LOC108334693 | PREDICTED: ATP synthase delta chain, chloroplastic [Vigna angularis] |
|  | gene-LOC108324248 | PREDICTED: uncharacterized protein LOC108324248 [Vigna angularis] |
|  | gene-LOC108337370 | PREDICTED: psbP-like protein 1, chloroplastic [Vigna angularis] |
| Photosynthesis antenna proteins | gene-LOC108337426 | PREDICTED: chlorophyll a-b binding protein AB96 isoform X2 [Vigna angularis] |
|  | gene-LOC108346034 | PREDICTED: chlorophyll a-b binding protein CP26, chloroplastic [Vigna angularis] |
|  | gene-LOC108343722 | PREDICTED: chlorophyll a-b binding protein of LHCII type 1-like [Vigna angularis] |
